# miRNA 17 Family Regulates Cisplatin-Resistant and Metastasis by Targeting TGFbetaR2 in NSCLC

**DOI:** 10.1371/journal.pone.0094639

**Published:** 2014-04-10

**Authors:** Zeyong Jiang, Jun Yin, Wenfan Fu, Yijun Mo, Youguang Pan, Lu Dai, Haoda Huang, Siwen Li, Jian Zhao

**Affiliations:** Department of Chest Surgery, Cancer Center of Guangzhou Medical University, Guangzhou, Guangdong, China; University of Bonn, Bonn-Aachen International Center for IT, Germany

## Abstract

MicroRNAs (miRNAs) have been proven to play crucial roles in cancer, including tumor chemotherapy resistance and metastasis of non-small-cell lung cancer (NSCLC). TGFβ signal pathway abnormality is widely found in cancer and correlates with tumor proliferation, apoptosis and metastasis. Here, miR-17, 20a, 20b were detected down-regulated in A549/DDP cells (cisplatin resistance) compared with A549 cells (cisplatin sensitive). Over-expression of miR-17, 20a, 20b can not only decrease cisplatin-resistant but also reduce migration by inhibiting epithelial-to-mesenchymal transition (EMT) in A549/DDP cells. These functions of miR-17, 20a, 20b may be caused at least in part via inhibition of TGFβ signal pathway, as miR-17, 20a, 20b are shown to directly target and repress TGF-beta receptor 2 (TGFβR2) which is an important component of TGFβ signal pathway. Consequently, our study suggests that miRNA 17 family (including miR-17, 20a, 20b) can act as *TGFβR2* suppressor for reversing cisplatin-resistant and suppressing metastasis in NSCLC.

## Introduction

Lung cancer, especially the non-small cancer lung cancer (NSCLC), is the most frequently malignant cancer [1], [2]. In NSCLC, the leading death cause is chemotherapy resistance and metastasis, yet the underlying mechanisms of them remain largely unclear [3]–[5]. Transforming growth factor-β1 (TGF-β1) can combine with the TGF-beta receptor 2 (TGFβR2) to activate the TGFβ signal pathway which has been suggested to be an important regulator of proliferation, apoptosis, epithelial-to-mesenchymal transition (EMT) and metastasis in various cancers [6]–[8]. It is well known that abnormal proliferation and apoptosis are important for the occurring of chemotherapy resistance in cancer therapy, and EMT is a key step in the progression of tumors toward invasion and metastasis [7], [8]. EMT is characterized by different regulations of epithelial and mesenchymal genes, for example, the increasing of mesenchymal markers vimentin and N-cadherin and the decreasing of epithelial marker E-cadherin are associated with EMT [9]–[13].

MicroRNAs (miRNAs) are small endogenous non-coding interfering RNA molecules involved in gene expression and lead to mRNA cleavage or translational repression through binding to the 3′-untranslated region (3′UTR) of target gene mRNA [14]. miRNAs often occur abnormal expression in human cancers and can act as essential modulators for carcinogenesis, chemotherapy resistance and metastasis [15]. Many papers have been reported that over-expressions of the miR-17-92 cluster (including miR-17, 18a, 19a, 20a, 19b-1, 92a-1) play important roles for the development of NSCLC [16], [17]. The miR-17-92 cluster also has been named as oncomir-1 associated with carcinogenesis and typically exhibit increased expression in tumors as a classical oncogene [18].

However, there are few reports about the roles of miR-17, 20a, 20b (miR-17, 20a belong to miR-17-92 cluster and miR-17, 20a, 20b are in the miRNA 17 family) in drug resistance and metastasis of NSCLC. In our present study, microarray profiles demonstrated the down-regulation of miR-17, 20a, 20b in A549/DDP cells (cisplatin resistance) compared to its parental A549 cells (cisplatin sensitive), and over-expression of miR-17, 20a, 20b could reduce cisplatin-resistant and migratory capability in A549/DDP cells. Subsequent experiments confirmed that miR-17, 20a, 20b could regulate the expression of *TGFβR2* at both mRNA and protein levels by directly binding with 3′UTR region of *TGFβR2*, so that these three miRNAs could inhibit *TGFβR2* that brings about cisplatin-resistant and migration. Our results suggest that miRNA 17 family (miR-17, 20a, 20b) play important roles in the regulation of cisplatin-resistant and migratory capability by targeting *TGFβR2* of TGFβ signal pathway. miRNA 17 family has the potential as key regulatory factors for the chemotherapy resistance and metastasis of NSCLC.

## Materials and Methods

### Cell lines and in vitro cisplatin-resistant model

Human A549 cell lines were obtained from Sun Yat-sen University. A549/DDP (cisplatin-resistant A549) cells were induced by using progressive concentration of cisplatin. Briefly, the A549 cells in logarithmic growth were treated with 0.5 μmol/L of cisplatin. After 48 h, cisplatin was withdrawn and cells were cultured without cisplatin until they recovered. Then, the same treatment was performed, and when the cells were resistant to the current concentration, the cisplatin concentration was gradually increased to 1–4 and finally to 6 μmol/L. When the induced cells survived in 6 μmol/L of cisplatin for about 2 months with a normal activity, the cells were confirmed to be cisplatin-resistant and named A549/DDP. Cells were cultured in RPMI1640 medium, supplemented with 10% fetal bovine serum (FBS) and maintained in a humidified incubator with 5% CO_2_ at 37°C.

### Microarray detection of miRNA expression

The total RNA of A549 cells and A549/DDP cells were isolated by Total RNA Purification Kit. The absorption value of RNA sample was examined at 230, 260 and 280 nm wavelength by tracing ultraviolet spectrophotometer to derive RNA concentrations. The A260/280 values of total RNA samples from A549 cells and A549/DDP cells were all between 1.80 and 2.00. The results of quality test showed that the total RNA samples of A549 cells and A549/DDP cells were allowed to compare the miRNA expression by microarray assay.

Microarray assay was performed using a service provider (LC Sciences). The hybridization assays were carried out in two experimental repeats of the RNA samples obtained from A549/DDP cells (Cy5-labeled) and A549 cells (Cy3-labeled). Hybridization images were collected using a laser scanner and digitized using Array-Pro image analysis software. Data were analyzed by first subtracting the background and then normalizing the signals using an LOWESS filter. For these two repeat experiments, the expression profiles of A549 cells and A549/DDP cells were compared with each other, and the ratios of these two sets of expression difference (log transformed, balanced) were indicated by colors from red (high expression) to green (low expression). The p-values of the t-test were calculated, and differentially detected signals were those with less than 0.01 p-values.

### Cytotoxicity assay

The cell-counting kit-8 (CCK-8, Dojindo Laboratories, Japan) colorimetric assay was used to measure the cell viability with triplicate experiments for each set of conditions according to the protocol of the manufacturer. Briefly, the suspensions of cells (transfected or untransfected) were incubated at a density of 3–5×10^3^ cells/well in 96-well plates and grew at 37°C with 7 different concentrations of cisplatin (0.25, 0.5, 0.75, 1.0, 1.25, 1.5, 2.0 μg/ml for A549 cells; 2.0, 4.0, 6.0, 8.0, 10.0, 12.0, 16.0 μg/ml for A549/DDP cells). After the indicated treatments (48 h), the medium with cisplatin were removed and RPMI1640 medium (Gibco, USA) of 90 μL and CCK-8 solution of 10 μL were added into each well of the plate. The cells on the plate were incubated for 3–6 h in the incubator. The absorbance at 450 nm wavelength was measured on an automated reader (TECAN, Switzerland). Control groups were cultivated under the same conditions without cisplatin. The viability of the cells was calculated as follows: 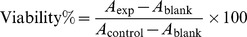



In which *A*_control_ is absorbance of control well, *A*_exp_ is absorbance of drug-treated well and *A*_blank_ is absorbance of the well containing medium and CCK-8 solution in the absence of cells.

Quantitative analysis of cisplatin resistance level was detected by CCK-8 assay and represented in IC50 (μg/ml).

### RNA extraction and RT-PCR

Total RNA was extracted by Trizol (Invitrogen, USA) according to the manufacturer's instruction. 1 μg RNA was reversely transcripted by reverse transcription kit (Takara, Japan), and SYBR Green Realtime PCR Master Mix (TOYOBO, Japan) to quantitative PCR was performed by using ABI ViiATM7Dx Real-Time PCR System (Life Technologies, USA). Expression levels of miRNA and mRNA were normalized by U6 and β-actin (Sigma, USA) separately.

Primer:

si-Notarget Control: 5′-UUCUCCGAACGUGUCACGUTT-3′

si-TGFβR2: 5′-GACCUCAAGAGCUCCAAUATT -3′

*TFGβR2*: Forward 5′-GTCGCTTTGCTGAGGTCTATAA-3′

Reverse 5′-CTCTGTCTTCCAAGAGGCATAC-3′

*E-cadherin*: Forward 5′-GCCAGGAAATCACATCCTACA-3′

Reverse 5′-TGGCAGTGTCTCTCCAAATC-3′

*Vimentin*: Forward 5′-CAGGAACAGCATGTCCAAATC-3′

Reverse 5′-GGCAGCCACACTTTCATATTG-3′

### Lipofectamine transfection assay

MiR-17, 20a, 20b mimics, miR-17, 20a, 20b inhibitors, si-TGFβR2 and their negative controls (all purchased from Sigma, USA) were transfected at a final concentration of 50 nM, 100 nM, 100 nM respectively by Lipofectamine RNAiMAX Reagent (Invitrogen, USA) according to the manufacturer's protocol. Firstly, Lipofectamine RNAiMAX Reagent and miRNA (siRNA) were diluted in Opti-MEM medium respectively. Then, diluted miRNA (siRNA) were mixed with diluted Lipofectamine RNAiMAX Reagent (1∶1 ratio) and incubated for 5 minutes. Finally, miRNA (siRNA)-Reagent mixture were added into fresh culture medium and incubated with cells for 24 h.

### Transwell migration assay

The migratory capability of A549 cells and A549/DDP cells (transfected or untransfected) was detected by using transwell-chamber culture systems (Becton Dickinson, USA). After 48 h of incubation at 37°C, cells were transferred into the upper chamber of the transwell with serum-free growth medium (1×10^5^ cells per well of 8 μm 24-well transwell). Complete growth medium containing 10% FBS was added to the lower chamber as a chemoattractant. Following 24 h of incubation at 37°C, cells on the upper surface of upper chamber (nonmigratory cells) were removed by cotton swabs, and cells on the lower surface of filters were fixed and stained with the Giemsa stain. The number of invaded cells was counted under light microscope (LAICA, Germany).

### Western Blotting

The cells were harvested at 72 h after transfection and lysed by RIPA buffer for 30 min at 4°C. 50 μg proteins were loaded into 15% SDS–PAGE for analysis. The first antibody was rabbit polyclonal anti-E-cadherinand, rabbit polyclonal anti-Vimentin and rabbit polyclonal anti-TGFβR2 respectively (all purchased by Cell Signaling USA, 1∶1000 dilutions). The secondary antibody was goat anti-rabbit IgG conjugated with HRP (horseradish peroxidase) with a dilution of 1∶10000. The bound antibodies were detected using ECL Plus Western Blotting Detection system (Life Technologies, USA).

### Dual luciferase activity assay

The 3′-UTR of human TGFβR2 containing the putative binding site for miR-17, 20a, 20b was chemically synthesized and inserted into psiCHECK-2 vector (Promega, USA) by using Endonuclease XhoI and NotI. 24 h prior to transfection, cells were plated at 8000 cells/well in 96-well plates. 50 ng psiCHECK-2-*TGFβR2*-3′UTR (wide type, WT; and mutant type, MUT) and 1.5 nmol miRNA mimics (controls) were co-transfected by Lipofectamine RNAiMAX Reagent (Invitrogen, USA) according to the manufacturer's instructions. Luciferase activity was measured at 48 h after co-transfection, using the Dual Luciferase Reporter Assay System (Promega, USA). Firefly luciferase activity was normalized to Renilla luciferase activity for each well. Three independent experiments were performed repeatedly.

*TGFβR2* WT: Forward 5′-GGCCTCGAGAGACGGCTCCCTAAACAC-3′

Reverse 5′-GGCGCGGCCGCGGAACACTGGTCCTCAAAC-3′

*TGFβR2* MUT: Forward 5′-CCAATAACATTTGCTGAATATTAATGCCTG-3′

Reverse 5′-CAGGCATTAATATTCAGCAAATGTTATTGG-3′

### Statistical analysis

All values of three repeated experiments were expressed as mean ± standard deviation (SD). RT-PCR, cytotoxicity and transwell assay were analyzed by one-way ANOVA (SPSS 15.0). IC50 value was assessed by profit regression analysis using SPSS15.0 statistical software. Difference was considered as significant if the probability was less than 0.05 (P<0.05).

## Results

### miR-17, 20a, 20b regulate cisplatin-resistant

**Figure 1:**
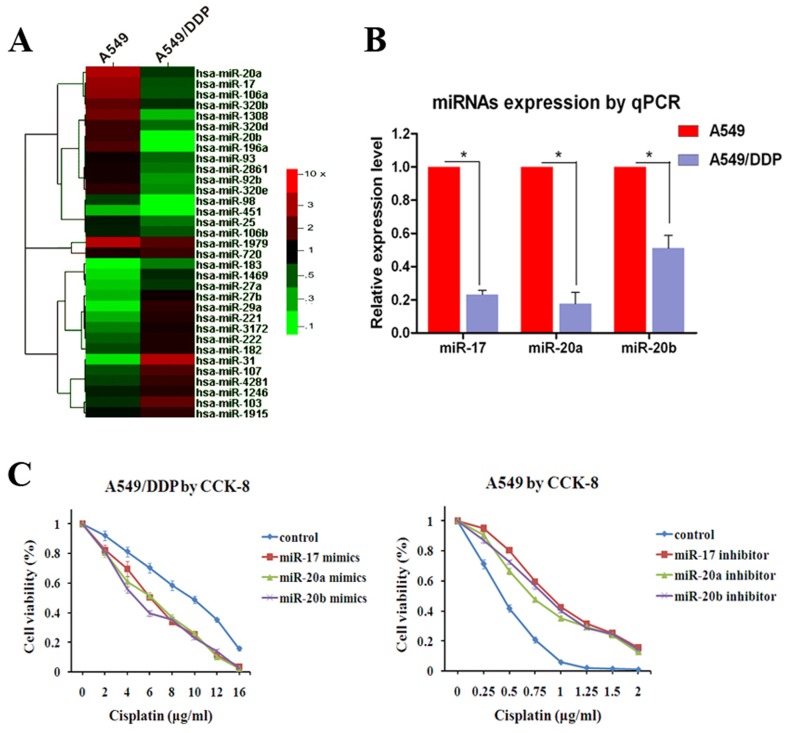
miR-17, 20a, 20b are low-expressed in A549/DDP cells and regulate cisplatin-resistant. (**A**) Clustering of cisplatin-resistant related miRNAs in A549 and A549/DDP cells. The vertical axis corresponds to the expression difference of miRNAs, A549 means A549 cells compared with A549/DDP cells in one microarray assay, A549/DDP means A549/DDP cells compared with A549 cells in another repeat microarray assay. The horizontal axis corresponds to miRNAs. miRNA expression levels were depicted as color variation from red (high expression) to green (low expression) according to color bar scale. (**B**) RT-PCR validated that miR-17, 20a, 20b expression levels were significantly lower in A549/DDP cells. (**C**) Over-expression or inhibition of miR-17, 20a, 20b significantly increased or decreased the growth-inhibitory effect of cisplatin in A549/DDP cells or A549 cells. *P<0.05. Results were representative of three experiments.

We compared the miRNA expression profiles of the cisplatin-resistant A549/DDP cells and its parental A549 cells by using miRNA microarray chip analysis, 33 miRNAs had statistical differences in expression between A549/DDP cells and A549 cells (Figure 1A). Table 1 lists the miRNAs with the highest differential expression levels in A549/DDP cells compared with A549 cells. Of note, several of the top differential expressed miRNAs (miR-17, miR-20a, miR-20b; fold change >2, P<0.001) contain the same seed sequence and were down-regulated in A549/DDP cells significantly. By using RT-PCR, we also validated the decreased expression levels of these three miRNAs in A549/DDP cells compared with A549 cells (Figure 1B). It was suggested that all these three miRNAs of the miRNA 17 family were closely related to the cisplatin resistance.

**Table 1:** Differential miRNA expression in A549/DDP cells compared with A549 cells.

miRNA ID	Fold Change (log2) (median)	P Value
hsa-miR-31	3.92	<0.001
**hsa-miR-20b**	−3.65	<0.001
hsa-miR-196a	−3.28	<0.001
hsa-miR-1308	−2.94	<0.001
hsa-miR-29a	2.73	0.001
**hsa-miR-20a**	−2.22	<0.001
**hsa-miR-17**	−2.22	<0.001
hsa-miR-221	2.04	0.001
hsa-miR-27b	1.94	0.002
hsa-miR-1469	1.87	0.002
hsa-miR-183	1.86	0.002
hsa-miR-320e	−1.82	0.003
hsa-miR-106a	−1.62	0.004
hsa-miR-92b	−1.60	0.004
hsa-miR-320d	−1.58	0.006
hsa-miR-107	1.52	0.005
hsa-miR-27a	1.51	0.005
hsa-miR-3172	1.42	0.008
hsa-miR-103	1.41	0.007
hsa-miR-2861	−1.35	0.007
hsa-miR-320b	−1.32	0.008
hsa-miR-222	1.17	0.007
hsa-miR-4281	1.10	0.007
hsa-miR-93	−1.06	0.007
hsa-miR-182	1.00	0.007

(Fold Change >1; P<0.01).

Note: Negative values represent down-regulation; positive values represent up-regulation.

To confirm the functional role of miR-17, 20a, 20b in cisplatin-resistant, we altered the expressions of these three miRNAs in A549/DDP and A549 cells by introducing miRNA-17, 20a, 20b mimics and miRNA-17, 20a, 20b inhibitors respectively. Over-expression of these three miRNAs decreased resistance of A549/DDP cells to cisplatin treatment (the IC50 of cisplatin decreased from 8.64 μg/ml to an average of 6.32 μg/ml, P<0.05), as evidenced by the growth inhibition curve (Figure 1C). Conversely, based on sequence similarities within their 6-nucleotide seed sequence, inhibition of any one miRNA could simultaneously suppress these three miRNAs and protected A549 cells from cisplatin treatment (the IC50 of cisplatin increased from 0.48 μg/ml to an average of 0.89 μg/ml, P<0.05) (Figure 1C). It is hypothesized that miR-17, 20a, 20b play important roles in cisplatin-resistant of NSCLC.

### miR-17, 20a, 20b regulate EMT and migration

**Figure 2:**
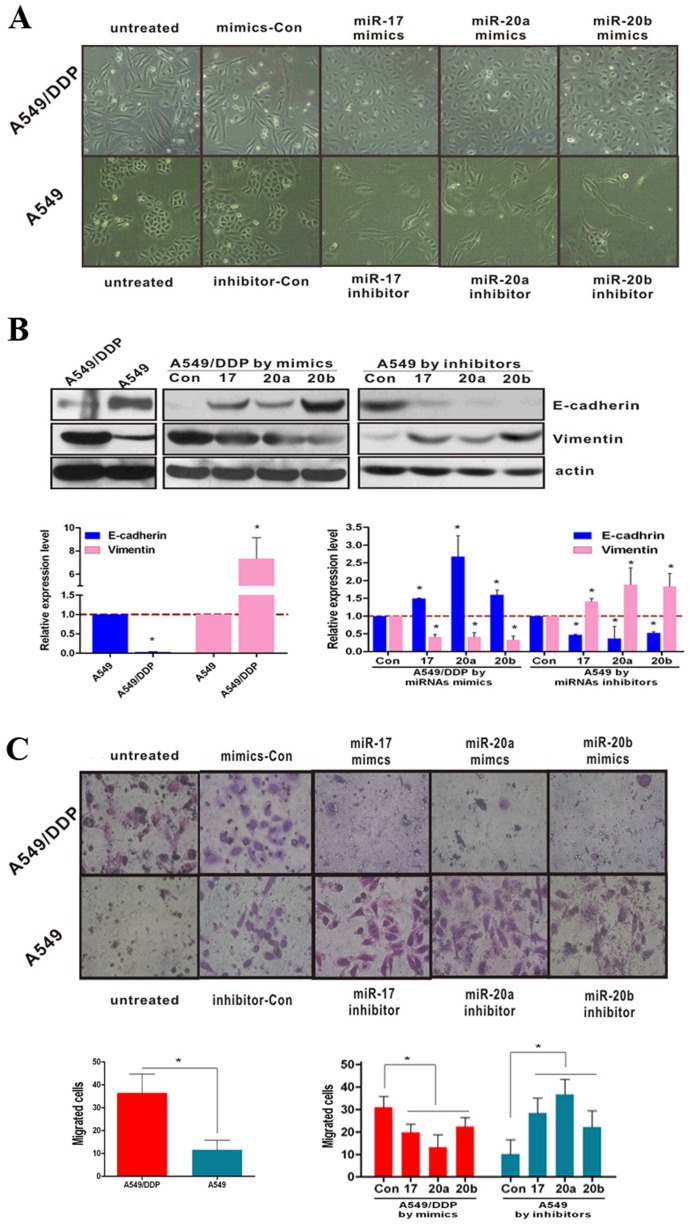
miR-17, 20a, 20b regulate EMT and migration. (**A**) Morphology of A549 and A549/DDP cells with or without miRNAs treatment. (**B**) mRNA and protein expression levels of E-cadherin and Vimentin in A549 and A549/DDP cells with or without miRNAs treatment were measured by RT-PCR and Western Blotting. (**C**) Transfer cells in migration assays were detected by transwell-chamber culture systems. Bar graphs show the number of migratory cells. *P<0.05. Results were representative of three experiments.

According to the cellular morphology, A549 cells exhibited the epithelial phenotype and A549/DDP cells showed the mesenchymal phenotype, suggested that EMT was occurred during the process of cisplatin-resistant (Figure 2A). To investigate the effect of miR-17, 20a, 20b on EMT, A549/DDP cells transfected with miR-17, 20a, 20b mimics could change to epithelial phenotype, and A549 cells transfected with miR-17, 20a, 20b inhibitors could change to mesenchymal phenotype (Figure 2A). Furthermore, the epithelial marker E-cadherin and the mesenchymal marker Vimentin were detected at mRNA and protein expression levels by RT-PCR and Western Blotting. A549/DDP cells transfected with miR-17, 20a, 20b mimics decreased Vimentin expression and increased E-cadherin expression, and A549 cells transfected with miR-17, 20a, 20b inhibitors showed an increase in Vimentin expression and a decrease in E-cadherin expression (Figure 2B).

We also found that the migratory capability of the A549/DDP cells exceed to A549 cells (Figure 2C). To further certify the role of miR-17, 20a, 20b in regulating migration, A549/DDP cells were transfected with miR-17, 20a, 20b mimics and A549 cells were transfected with miR-17, 20a, 20b inhibitors to perform transwell migration assay. Results showed that over-expression of miR-17, 20a, 20b could increase migration in A549/DDP cells and inhibition of them could reduce migration in A549 cells (Figure 2C). These results indicated that miR-17, 20a, 20b could regulate the metastasis efficiently in NSCLC.

### miRNA-17, 20a, 20b directly target and suppress *TGFβR2*

**Figure 3:**
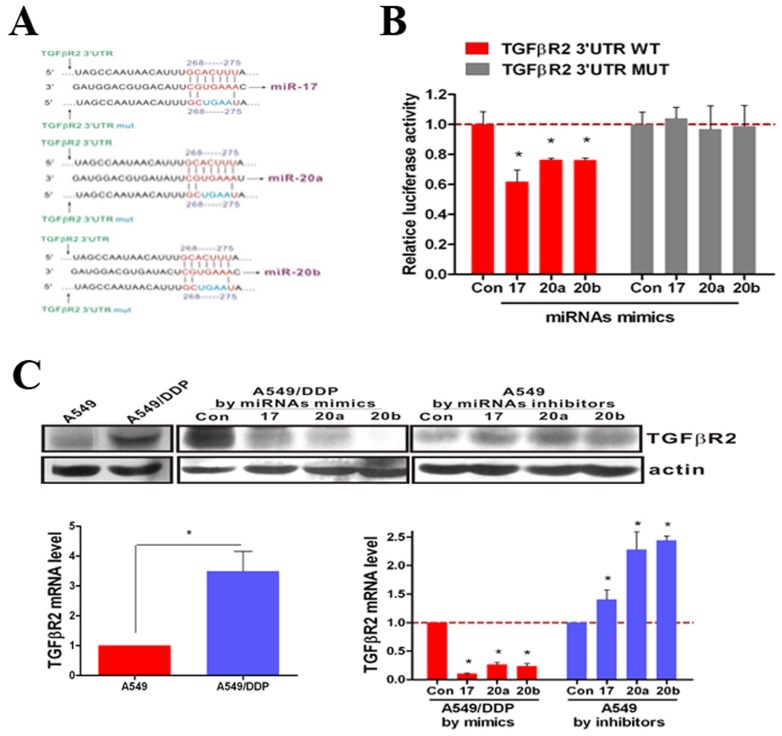
miR-17, 20a, 20b target TGFβR2 by directly binding to the TGFβR2 mRNA 3′-UTR. (**A**) Site-directed mutagenesis targeting potential miR-17, 20a, 20b binding sites (MUT) on the TGFβR2 mRNA 3′-UTR-luciferase construct. (**B**) Luciferase activities significantly decreased in the WT TGFβR2 mRNA 3′-UTR-luciferase plasmid transfected A549 cells after transfection of miR-17, 20a, 20b mimics. Effect was blocked in the MUT plasmid transfected A549 cells. (**C**) By RT-PCR and Western Blotting, the expression of *TGFβR2* increased significantly in A549/DDP cells compared with A549 cells. The expression of *TGFβR2* decreased in A549/DDP cells after transfection of miR-17, 20a, 20b mimics and increased in A549 cells after transfection of miR-17, 20a, 20b inhibitors. *P<0.05. Results were representative of three experiments.

By using targetscan prediction system, we identified a number of potential miR-17, 20a, 20b targets. Among the predicted miR-17, 20a, 20b targets, these three miRNAs are in the 3′UTR region of *TGFβR2*, a central receptor of the TGFβ signal pathway. To confirm that miR-17, 20a, 20b can regulate *TGFβR2* expression by directly binding to the *TGFβR2* 3′UTR, we generated luciferase reporter constructs containing specific mutations at putative miR-17, 20a, 20b binding site (Figure 3A). As shown in Figure 3B, when A549 cells were transfected with the wild type *TGFβR2* 3′UTR, co-transfection of miR-17, 20a, 20b mimics inhibited luciferase activity. In contrast, the effects of miR-17, 20a, 20b mimics were eliminated in A549 cells transfected with the mutant type *TGFβR2* 3′UTR. These results suggested that miR-17, 20a, 20b binds directly to putative *TGFβR2* 3′UTR regions, as predicted by the in silico model.

Moreover, we found that both the mRNA and protein expression levels of *TGFβR2* were up-regulated in A549/DDP cells compared with A549 cells (Figure 3C). As shown in Figure 3C, the expression levels of *TGFβR2* decreased in A549/DDP cells transfected with miR-17, 20a, 20b mimics and increased in A549 cells transfected with miR-17, 20a, 20b inhibitors. Thus, the expression of *TGFβR2* was enhanced when cisplatin-resistant occurs and regulated by miR-17, 20a, 20b.

### Suppression of *TGFβR2* expression reduces cisplatin-resistant and migration

**Figure 4:**
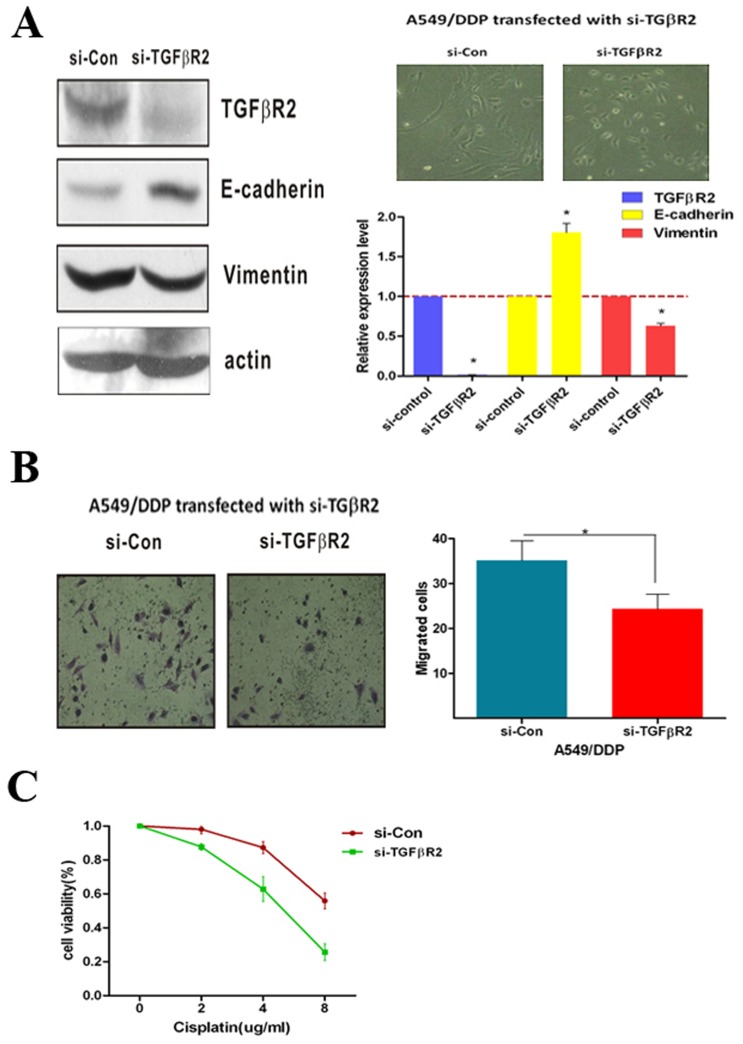
TGFβR2 silenced restrain cisplatin-resistant and reduce migration in A549/DDP cells. (**A**) After inhibition of TGFβR2 in A549/DDP cells by transfected with si-TGFβR2, the EMT phenotype changed evidenced by over-expression of E-cadherin and down-regulation of Vimentin, and (**B**) the migratory capability also significantly decreased. Bar graphs show the number of migratory cells. (**C**) TGFβR2 silenced induced by siRNA led to decrease cisplatin resistance, as shown by a significantly growth inhibition curve of cisplatin in A549/DDP cells. *P<0.05. Results were representative of three experiments.

To further confirm that *TGFβR2* was the target gene of miR-17, 20a, 20b to regulate cisplatin-resistant and metastasis, the small interfering RNA (siRNA) si-TGFβR2 was used to generate A549/DDP si-TGFβR2 cells which TGFβR2 was silenced. *TGFβR2* mRNA and protein expressions were significantly down-regulated in A549/DDP si-TGFβR2 cells, resulting in the inhibition of EMT and decrease of migratory capability (Figure 4A and 4B). Moreover, A549/DDP si-TGFβR2 cells were more sensitive to cisplatin-induced growth inhibition than A549/DDP cells, as reflected by the growth inhibition curve (Figure 4C). These results indicated that inhibition of *TGFβR2* in A549/DDP cells resulted in restrain cisplatin-resistant and reduced migration.

## Discussion

Based on the GLOBOCAN 2008 estimates, about 12.7 million cancer cases and 7.6 million cancer deaths were estimated to have occurred in 2008. Lung cancer is the leading cancer site in males, comprising 17% of the total new cancer cases and 23% of the total cancer deaths, and 85% of lung cancer are NSCLC [19], [20]. Surgical is the most effective treatment for NSCLC patients, others have to choose drug chemotherapy or radiation therapy. As one of the first-line chemotherapeutic agents for the treatment of NSCLC, cisplatin is a platinum-based compound that forms intra- and inter-strand adducts with DNA [22], [23]. However, almost around 80% of patients are cisplatin-resistant to the treatment of DDP-based chemotherapy regimens, and some substantial efforts have been made to clarify the mechanisms of cisplatin-resistant, such as deficiencies in DNA damage repair. For some patients, even if they are sensitive to chemotherapy in the beginning, acquired cisplatin-resistant will eventually occur [21]. In this study, we established the cisplatin-resistant strains A549/DDP cells by cisplatin inducing to research this type of acquired cisplatin-resistant, and it could be useful to explore general drug resistance mechanisms if we wanted to improve survival in NSCLC patients.

Our study established a correlation of miRNA 17 family with cisplatin-resistant and tumor metastasis, and this correlation likely was associated with the TGFβ signal pathway-mediated mechanism. Firstly, by miRNA microarray profiling, miR-17, 20a, 20b were down-regulation in cisplatin-resistant A549/DDP cells compared with A549 cells. Secondly, inhibition of miR-17, 20a, 20b increased cisplatin-resistant and migration of A549 cells, and over-expression of miR-17, 20a, 20b decreased cisplatin-resistant and migration of A549/DDP cells [6], [12]. Thirdly, miR-17, 20a, 20b blunted the TGFβ signal pathway by directly inhibiting its important component *TGFβR2*. Finally, *TGFβR2* silenced led to cisplatin sensitivity and migration inhibition in A549/DDP cells. In a word, our findings indicated that miRNA 17 family could regulate cisplatin-resistant and metastasis through TGFβR2-mediated mechanism that had not been previously reported in NSCLC [24], [25].

**Figure 5:**
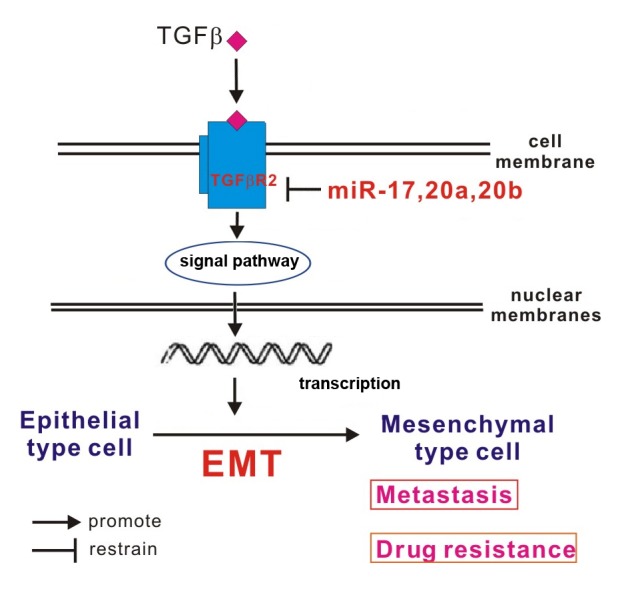
miR-17 family regulate cisplatin-resistant and metastasis by targeting TGFβR2 in NSCLC. TGFβ signal pathway can induce epithelial cells into mesenchymal cells by EMT, and mesenchymal cells show the potential of drug resistance and metastasis. In this study, miR-17, 20a, 20b can inhibit the TGFβ signal pathway by targeting TGFβR2, consequently, inhibition of miR-17, 20a, 20b can induce EMT and lead to cisplatin-resistant and migration in A549/DDP cells.

miR-17-92 cluster (including miR-17, 18a, 19a, 20a, 19b-1, 92a-1) usually is over-expression in malignant tumor and involved in carcinogenesis as the classical oncogene [15], [26]–[28]. On the other side, miR-17-92 cluster also can negatively regulate the oncogene *E2F1* or *MYC* to inhibit cell cycle progression as the tumor suppressor gene [29]. Another paper supports this viewpoint by exhibiting that miR-17, 20a can inhibit the metastasis in oral squamous cell carcinoma, suggesting that miRNA 17 family (including miR-17, 20a) may be the potential tumor suppressor gene too (miRNAs in the same family may have the same function) [30]. TGFβ signal pathway constitute of TGFβ, TGFβR, Smads protein family and some transcriptional regulatory factors. TGFβ has several subtypes and the TGF-β1 is most important one in human being [31]. TGFβR including three subtypes TβR-I, TβR-II, TβR-III but only TβR-II (TGFβR2) can accept TGF-β1 independently, the other two receptors cannot take a proper functioning unless TGFβR2 existing [32]. Several previous studies have suggested that TGFβ can activate the special signaling pathways to induce EMT and result in drug resistance and metastasis of cancer [6], [33]. Therefore, as shown in our results, EMT also can be induced by activation of TGFβ signal pathway to result in drug resistance and metastasis in NSCLC, and inhibition of *TGFβR2* by miRNA 17 family can interdict EMT to enhanced chemotherapy sensitivity and metastasis inhibition of NSCLC (Figure 5).

In this study, we specifically identified the correlation of miRNA 17 family with cisplatin-resistant and metastasis through TGFβ signal pathway-mediated mechanism in NSCLC. For this acquired cisplatin-resistant model, low-expression of miR-17, 20a, 20b is a critical factor in activating TGFβ signal pathway and inducing EMT by which A549/DDP cells increase the cisplatin-resistant and migration. In summary, our findings have the significant translational potential for identifying novel approaches to overcome chemotherapy resistance and metastasis, and the miR-17 family has the potential as a predictive biomarker for cisplatin-based therapy regimens and tumors metastasis in NSCLC.
