# Expression of Concern: miRNA 17 Family Regulates Cisplatin-Resistant and Metastasis by Targeting TGFbetaR2 in NSCLC

**DOI:** 10.1371/journal.pone.0222896

**Published:** 2019-09-19

**Authors:** The PLOS ONE Editors

Following publication, concerns were raised about duplicate images in Figs 2, 3 and 4 of this article [1].

Specifically, in Fig 2A the top left two panels have a region of overlap and have been taken from the same microscopy image. In Fig 3C “A549/DDP by miRNAs mimics” actin panel lanes 20a and 20b and in Fig 4A actin panel, the same image was used to represent the actin controls from two different experiments.

The corresponding author has stated these were mistakes in the preparation of the original figures and provided revised figures.

In Fig 2A, the “A549/DDP mimics-Con” panel was incorrect and has been replaced. Please see the correct Fig 2 and caption here.

**Fig 2:**
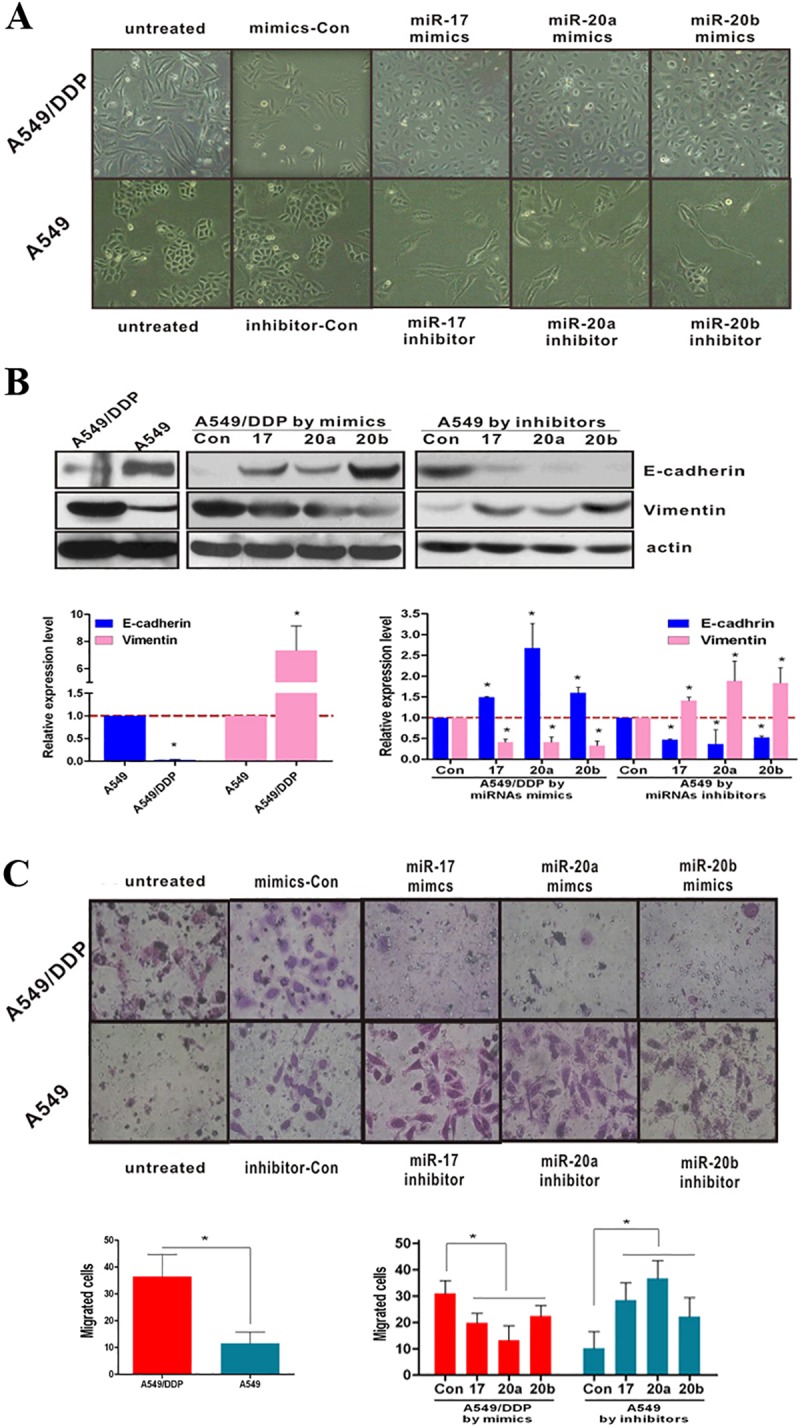
miR-17, 20a, 20b regulate EMT and migration. (**A**) Morphology of A549 and A549/DDP cells with or without miRNAs treatment. (**B**) mRNA and protein expression levels of E-cadherin and Vimentin in A549 and A549/DDP cells with or without miRNAs treatment were measured by RT-PCR and Western Blotting. (**C**) Transfer cells in migration assays were detected by transwell-chamber culture systems. Bar graphs show the number of migratory cells. *P<0.05. Results were representative of three experiments.

In Fig 3C, the “A549/DPP by mRNAs mimics” actin panel was incorrect and has been replaced. Please see the correct Fig 3 and caption here.

**Fig 3:**
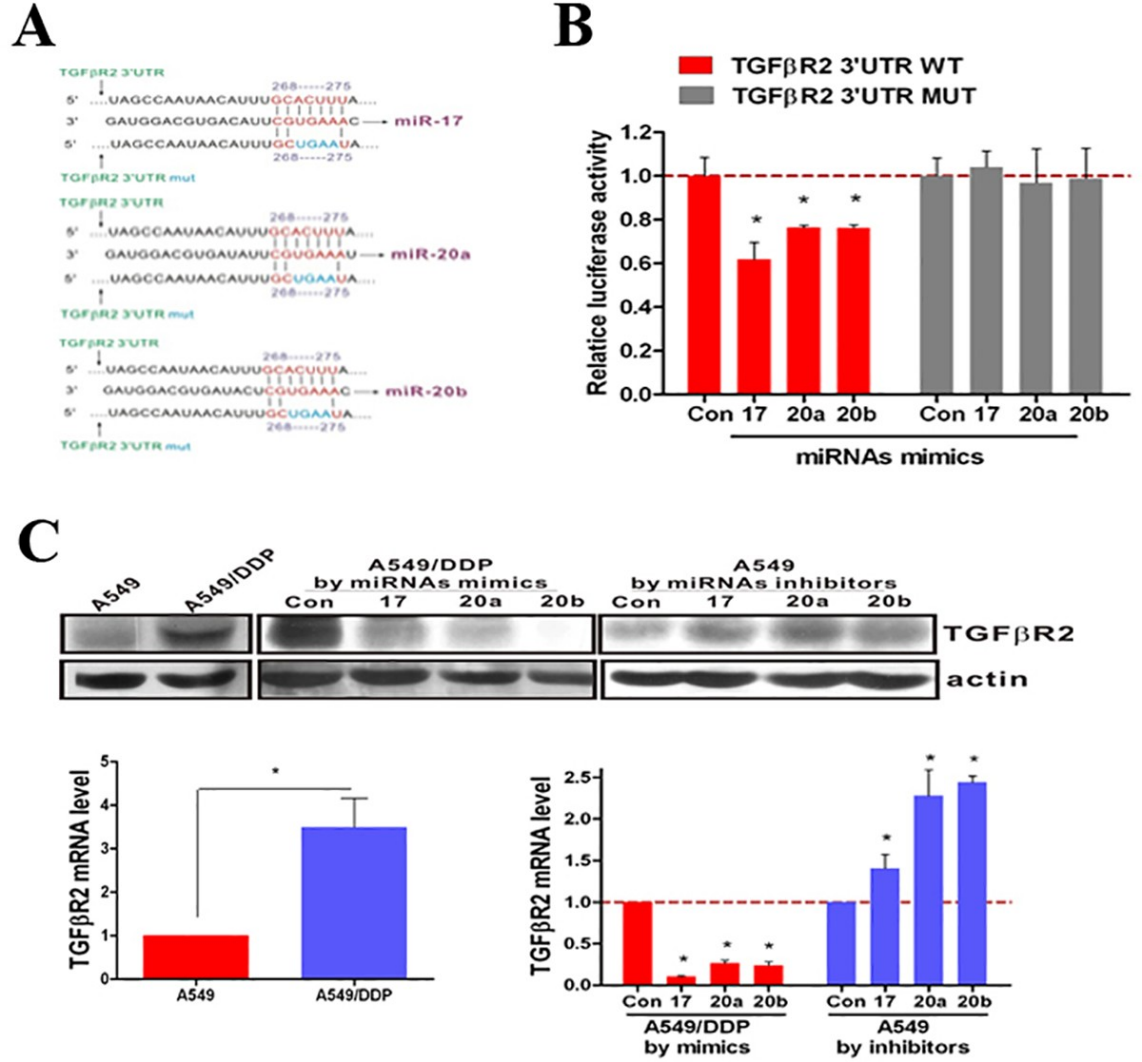
miR-17, 20a, 20b target TGFβR2 by directly binding to the TGFβR2 mRNA 3′-UTR. (**A**) Site-directed mutagenesis targeting potential miR-17, 20a, 20b binding sites (MUT) on the TGFβR2 mRNA 3′-UTR-luciferase construct. (**B**) Luciferase activities significantly decreased in the WT TGFβR2 mRNA 3′-UTR-luciferase plasmid transfected A549 cells after transfection of miR-17, 20a, 20b mimics. Effect was blocked in the MUT plasmid transfected A549 cells. (**C**) By RT-PCR and Western Blotting, the expression of *TGFβR2* increased significantly in A549/DDP cells compared with A549 cells. The expression of *TGFβR2* decreased in A549/DDP cells after transfection of miR-17, 20a, 20b mimics and increased in A549 cells after transfection of miR-17, 20a, 20b inhibitors. *P<0.05. Results were representative of three experiments.

In Fig 4A, the actin panel was incorrect and has been replaced. Please see the correct Fig 4 and caption here.

**Fig 4:**
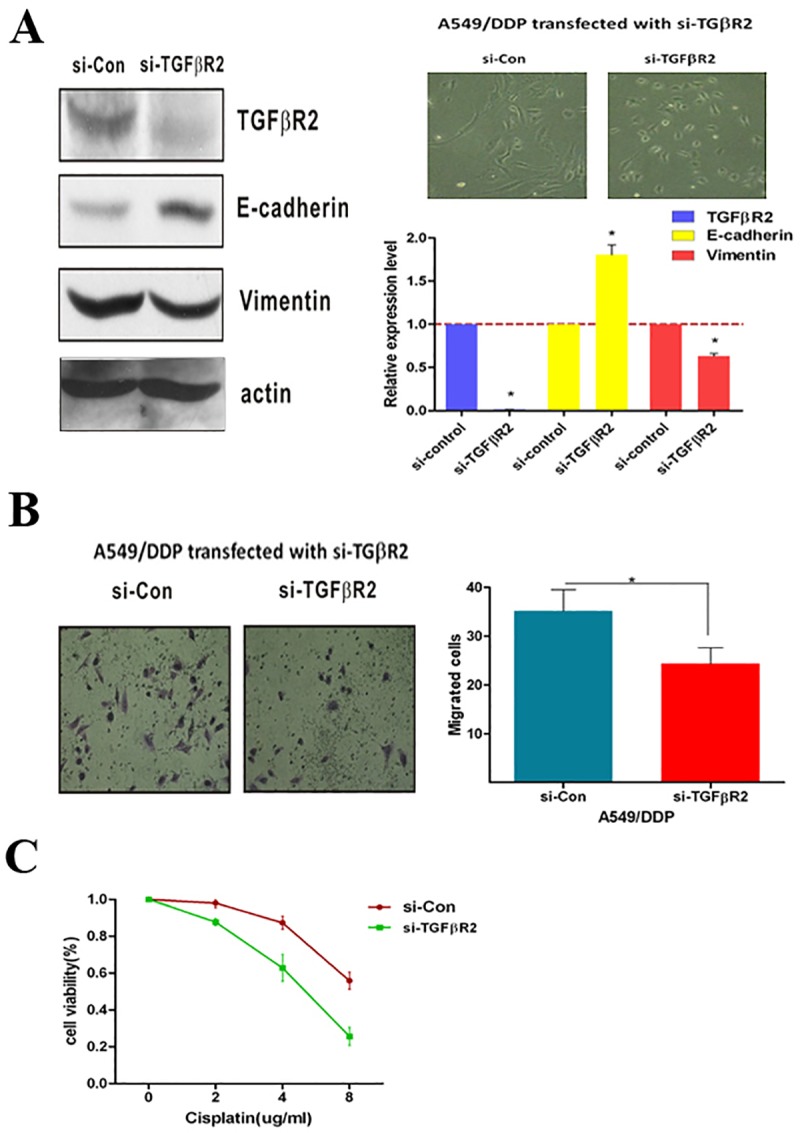
TGFβR2 silenced restrain cisplatin-resistant and reduce migration in A549/DDP cells. (**A**) After inhibition of TGFβR2 in A549/DDP cells by transfected with si-TGFβR2, the EMT phenotype changed evidenced by over-expression of E-cadherin and down-regulation of Vimentin, and (**B**) the migratory capability also significantly decreased. Bar graphs show the number of migratory cells. (**C**) TGFβR2 silenced induced by siRNA led to decrease cisplatin resistance, as shown by a significantly growth inhibition curve of cisplatin in A549/DDP cells. *P<0.05. Results were representative of three experiments.

The underlying blot images, microscopy images, and the data points underlying the charts in Figs 2, 3 and 4 are provided as Supporting Information, with the exception of the following items that are no longer available:

- The underlying blots for Fig 2B left hand panel E-cadherin, right hand panel “A549 by inhibitors” Vimentin and actin, and middle panel “A549/DPP by mimics” Vimentin.
- The underlying blot for Fig 2B middle panel “A549/DPP by mimics” E-caderin. A blot from a replicate experiment carried out at the time of the original study is included in the Supporting Information.
- The datapoints underlying the chart in Fig 4A, which shows mRNA levels detected by RT-PCR.

The authors have also provided the following clarifications and corrections:

- The samples used for actin and target protein detection were loaded into separate gels in the same order and marked on the corners of the gel and PVDF membrane, and for this reason, actin control bands appear in different blots to those of the target proteins.
- There is an error in the seventh sentence of the “miR-17, 20a, 20b regulate EMT and migration” subsection of the Results. The correct sentence is:Results showed that over-expression of miR-17, 20a, 20b could reduce migration in A549/DDP cells and inhibition of them could increase migration in A549 cells (Fig 2C).
- In the second sentence of the second paragraph of the Discussion section, references 6 and 12 are incorrectly placed and should not be included. The correct sentence is:Secondly, inhibition of miR-17, 20a, 20b increased cisplatin-resistant and migration of A549 cells, and over-expression of miR-17, 20a, 20b decreased cisplatin-resistant and migration of A549/DDP cells.

The underlying data for Fig 1 are not available. This includes data from a microarray experiment performed by service provider LC Sciences. The Data Availability Policy in effect at the time of submission of the article required public deposition of microarray data, which is in accordance with standard practice in the field at the time.

The *PLOS ONE* Editors issue this Expression of Concern to alert readers to the concerns about the unavailability of the microarray data, as well as the outlined concerns regarding the originally published images.

## Supporting information

S1 File Underlying image and data files for Figs 2B, 2C, 3B, 4B and 4C. (RAR)

S2 File Underlying image and data files for Figs 2A, 3C and 4A. (ZIP)
