# Supplementary figures and images for: Expression of Concern: miRNA 17 Family Regulates Cisplatin-Resistant and Metastasis by Targeting TGFbetaR2 in NSCLC
Source: PLoS One. 2019 Sep 19;14(9):e0222896. doi: 10.1371/journal.pone.0222896 (PMC6752771; doi:10.1371/journal.pone.0222896)

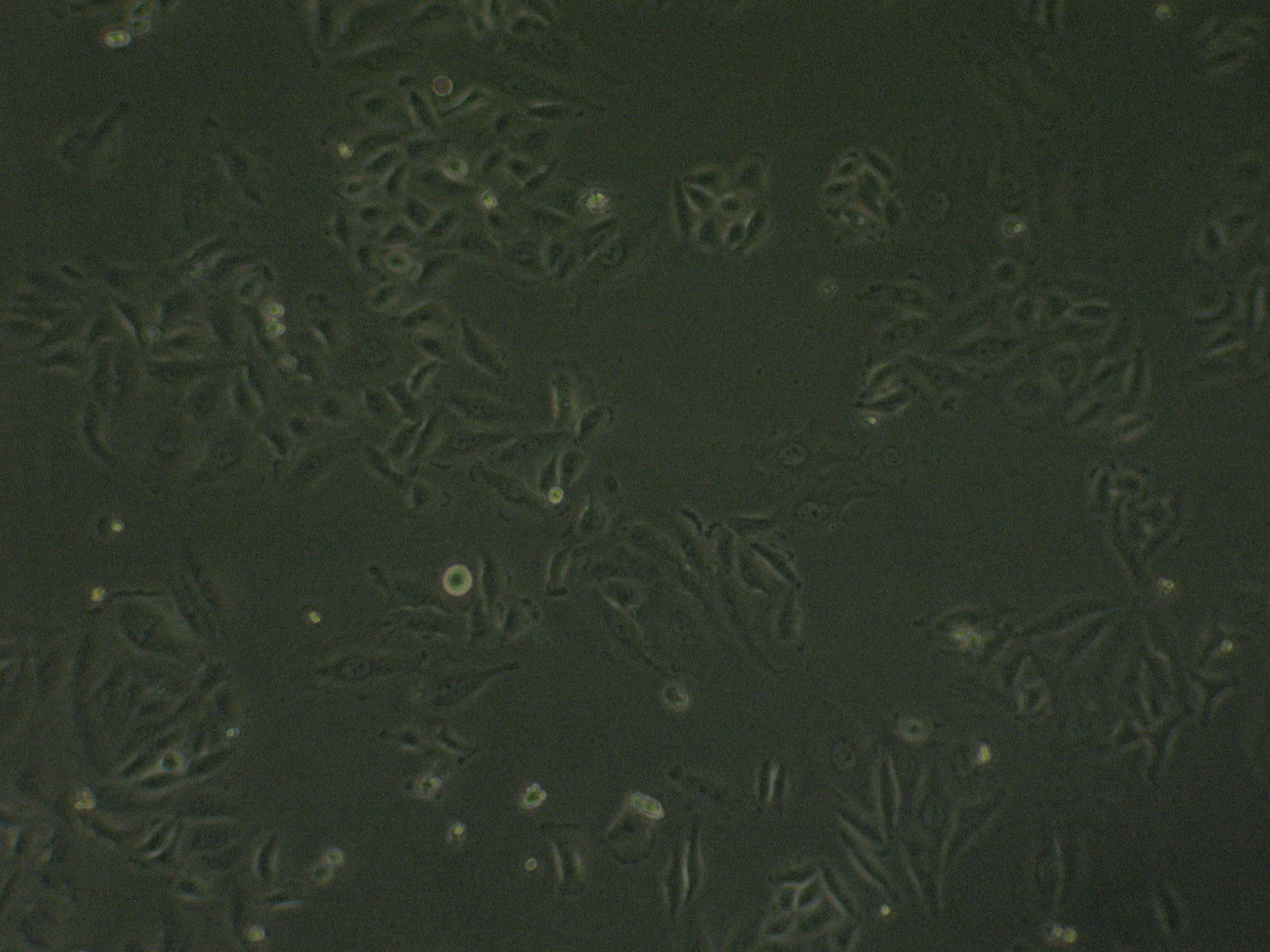

Supplement: S2 File — (ZIP) [file pone.0222896.s002.zip › S2_File/Figure 2A/A549 DDP/mimics-Con.JPG]

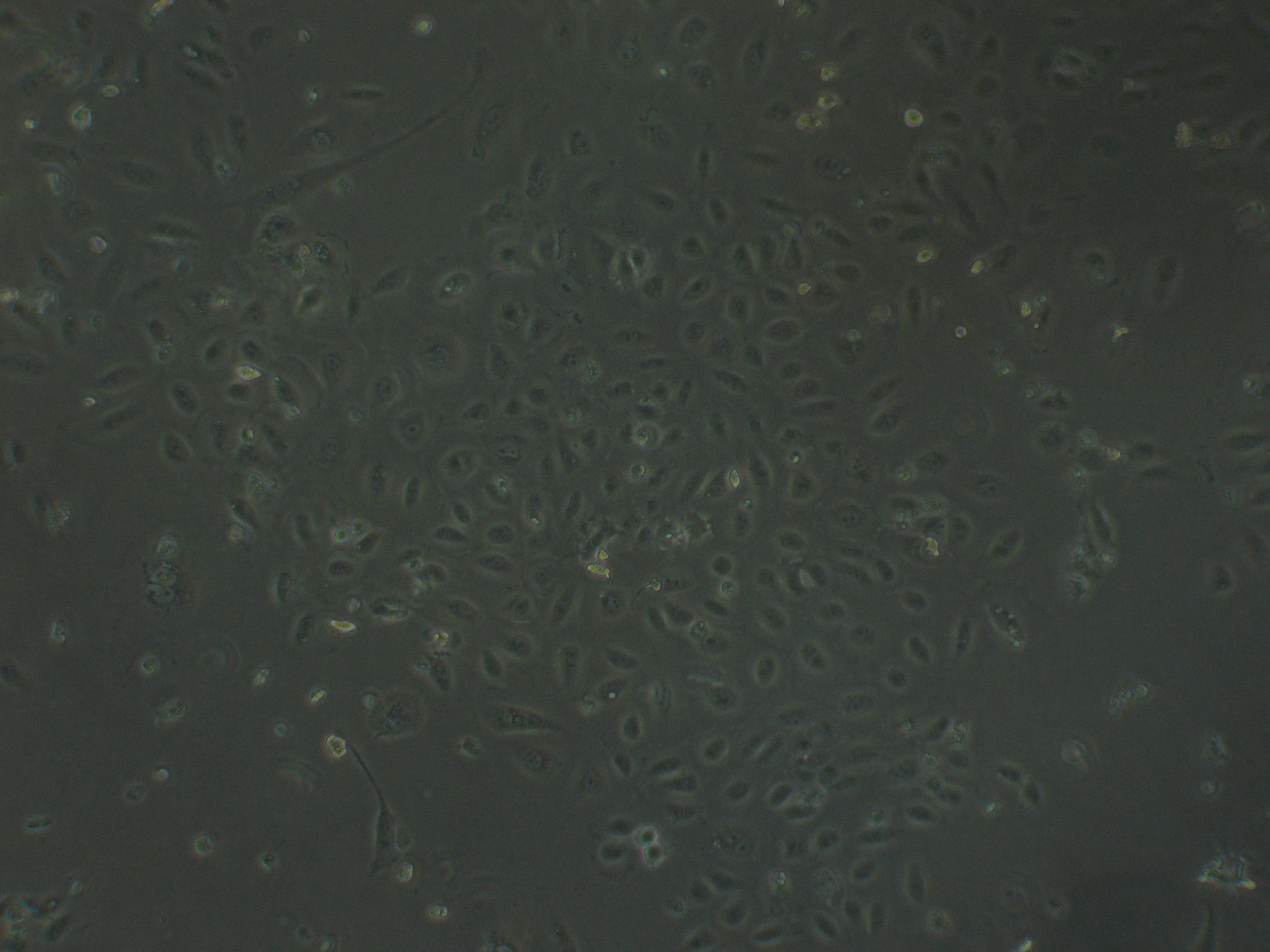

Supplement: S2 File — (ZIP) [file pone.0222896.s002.zip › S2_File/Figure 2A/A549 DDP/miR-17 mimics.JPG]

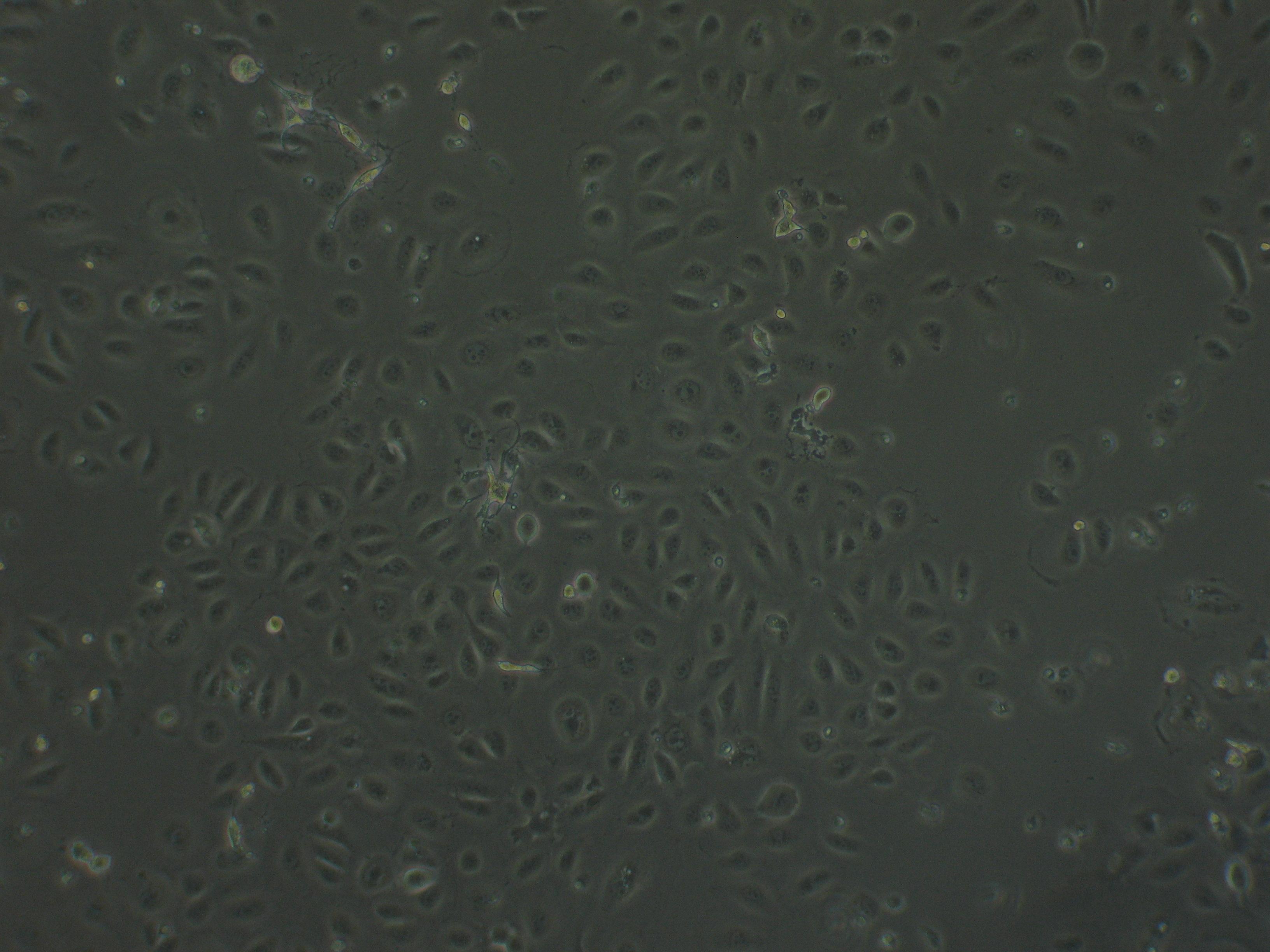

Supplement: S2 File — (ZIP) [file pone.0222896.s002.zip › S2_File/Figure 2A/A549 DDP/miR-20a mimics.JPG]

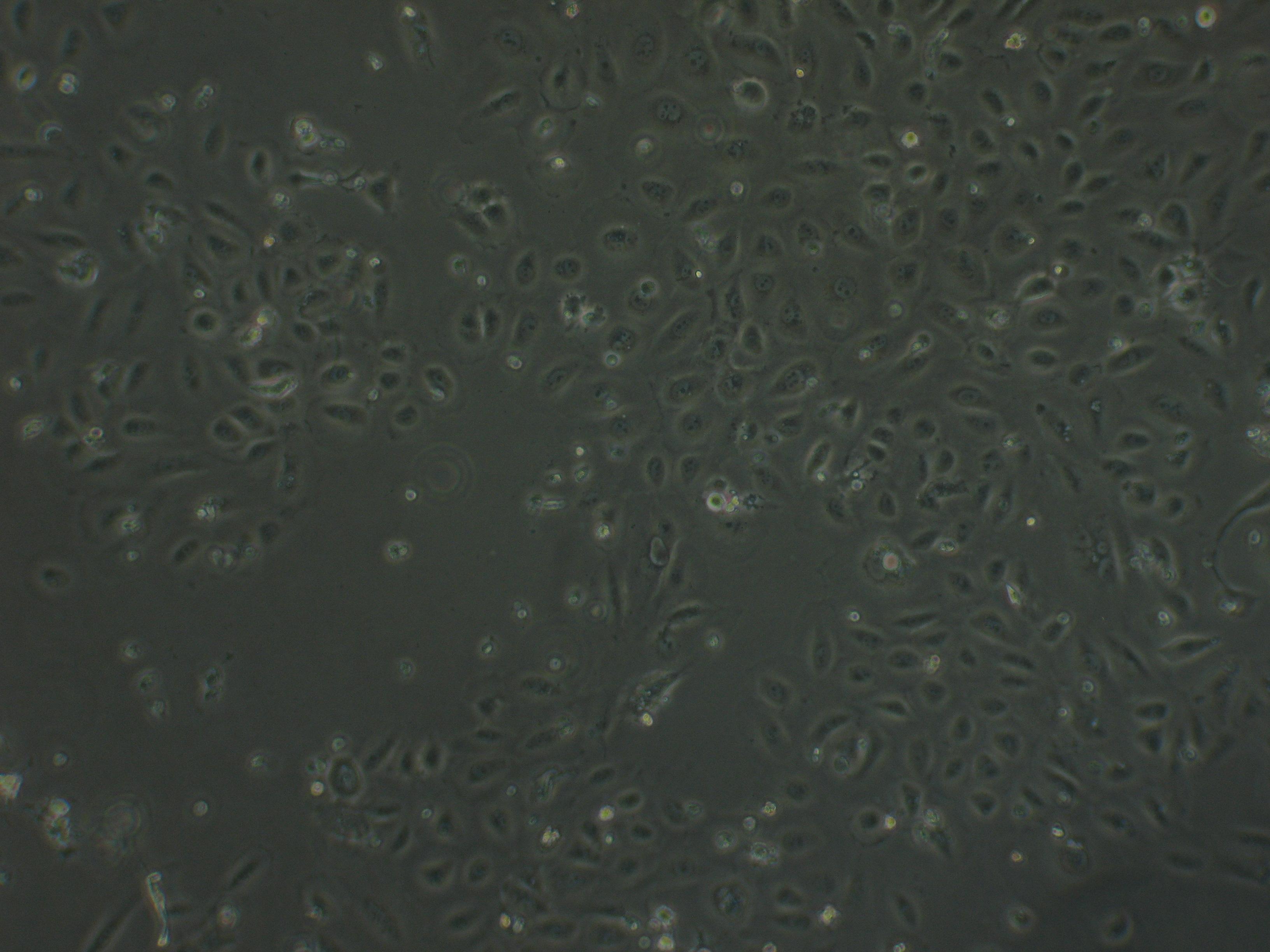

Supplement: S2 File — (ZIP) [file pone.0222896.s002.zip › S2_File/Figure 2A/A549 DDP/miR-20b mimics.JPG]

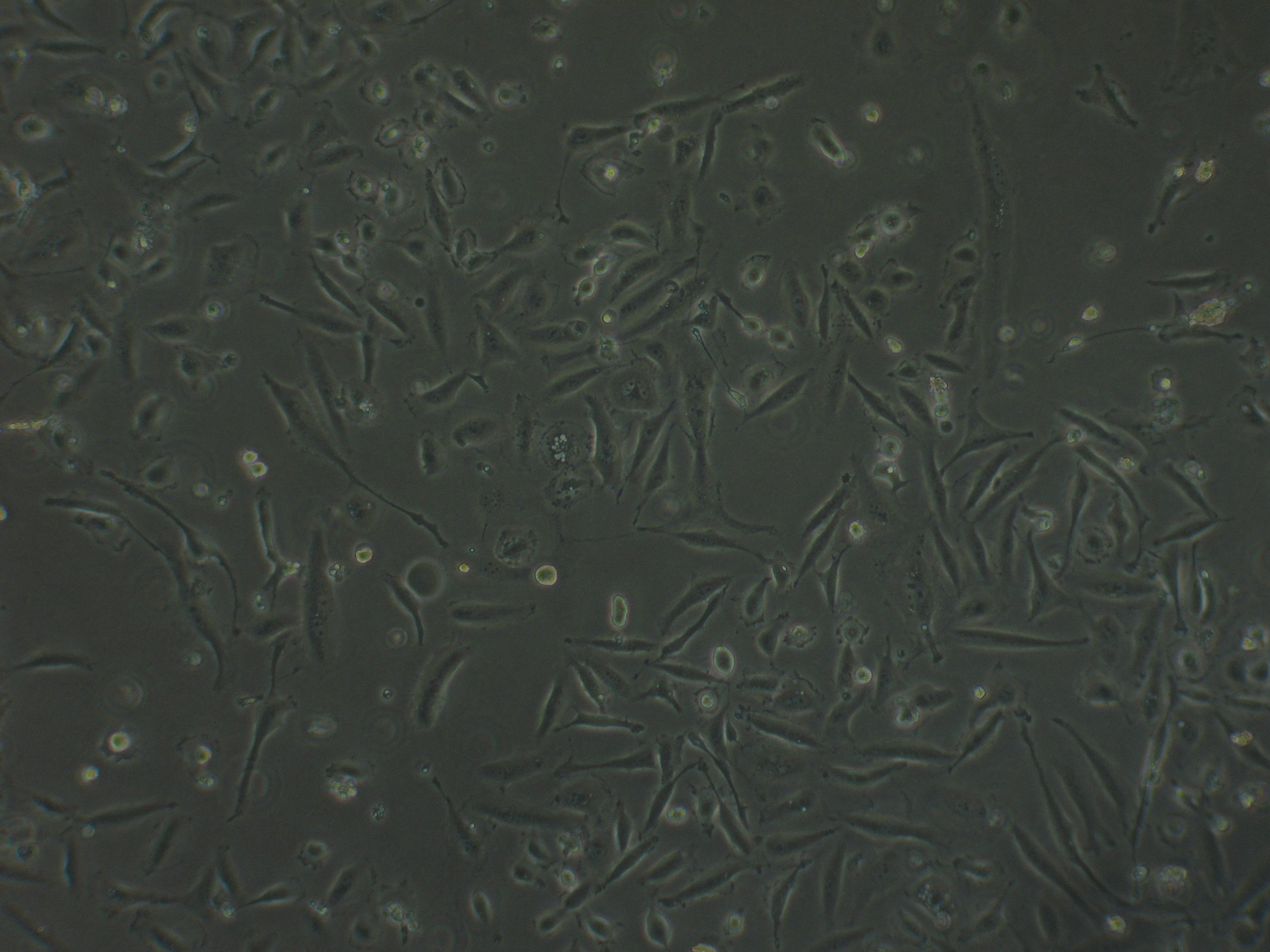

Supplement: S2 File — (ZIP) [file pone.0222896.s002.zip › S2_File/Figure 2A/A549 DDP/untreated.JPG]

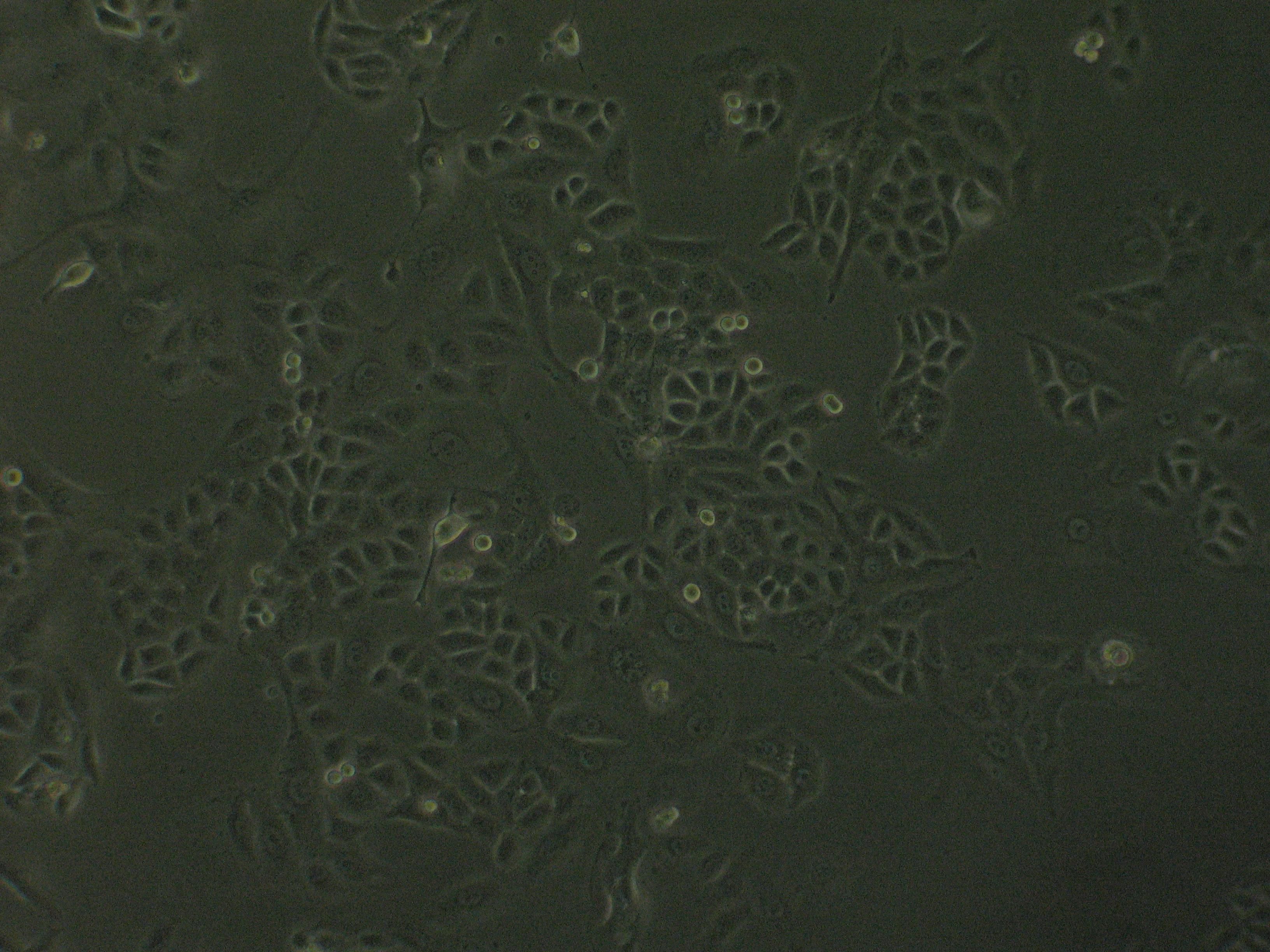

Supplement: S2 File — (ZIP) [file pone.0222896.s002.zip › S2_File/Figure 2A/A549/inhibitor-Con.JPG]

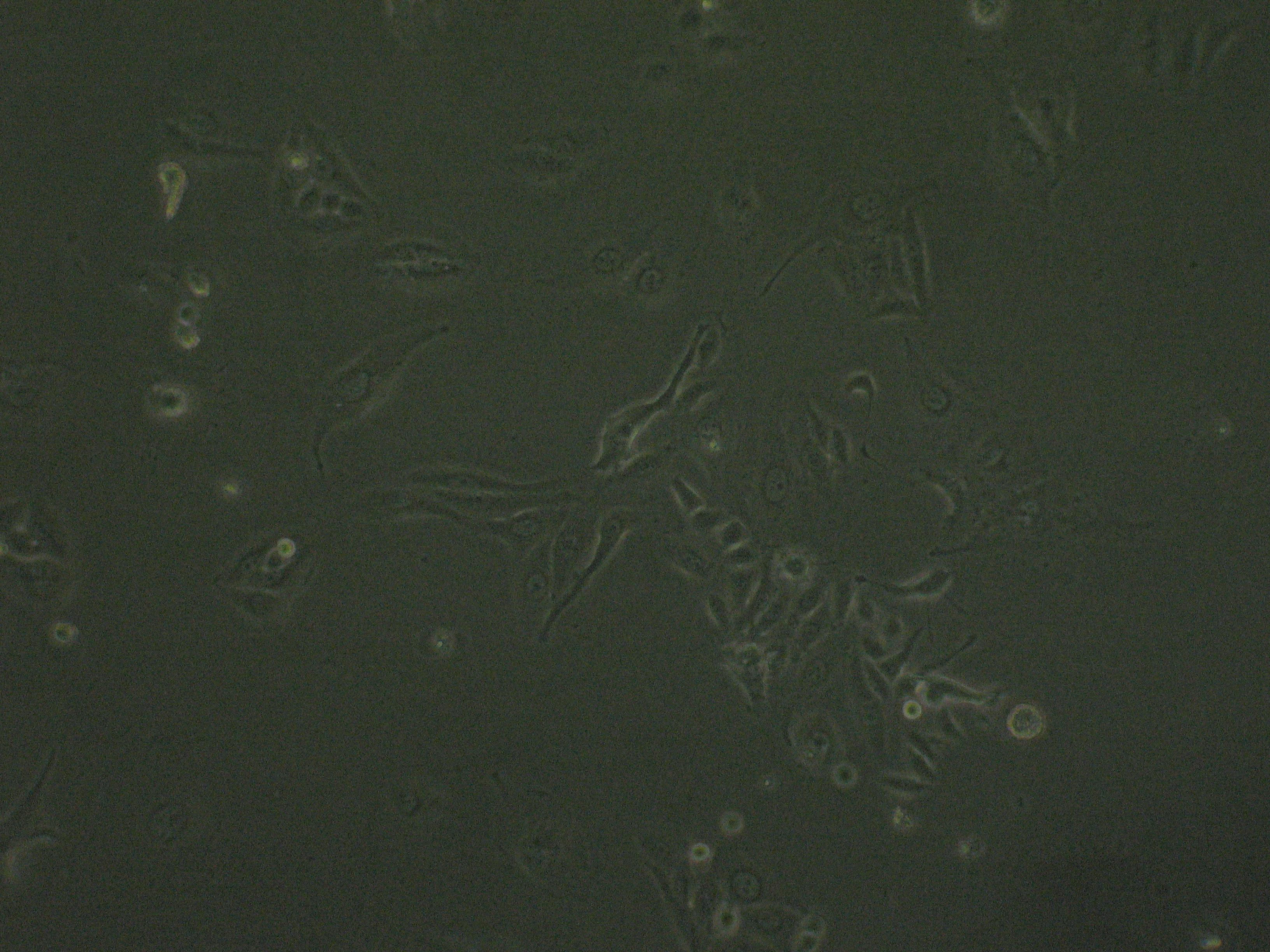

Supplement: S2 File — (ZIP) [file pone.0222896.s002.zip › S2_File/Figure 2A/A549/miR-17 inhibitor.JPG]

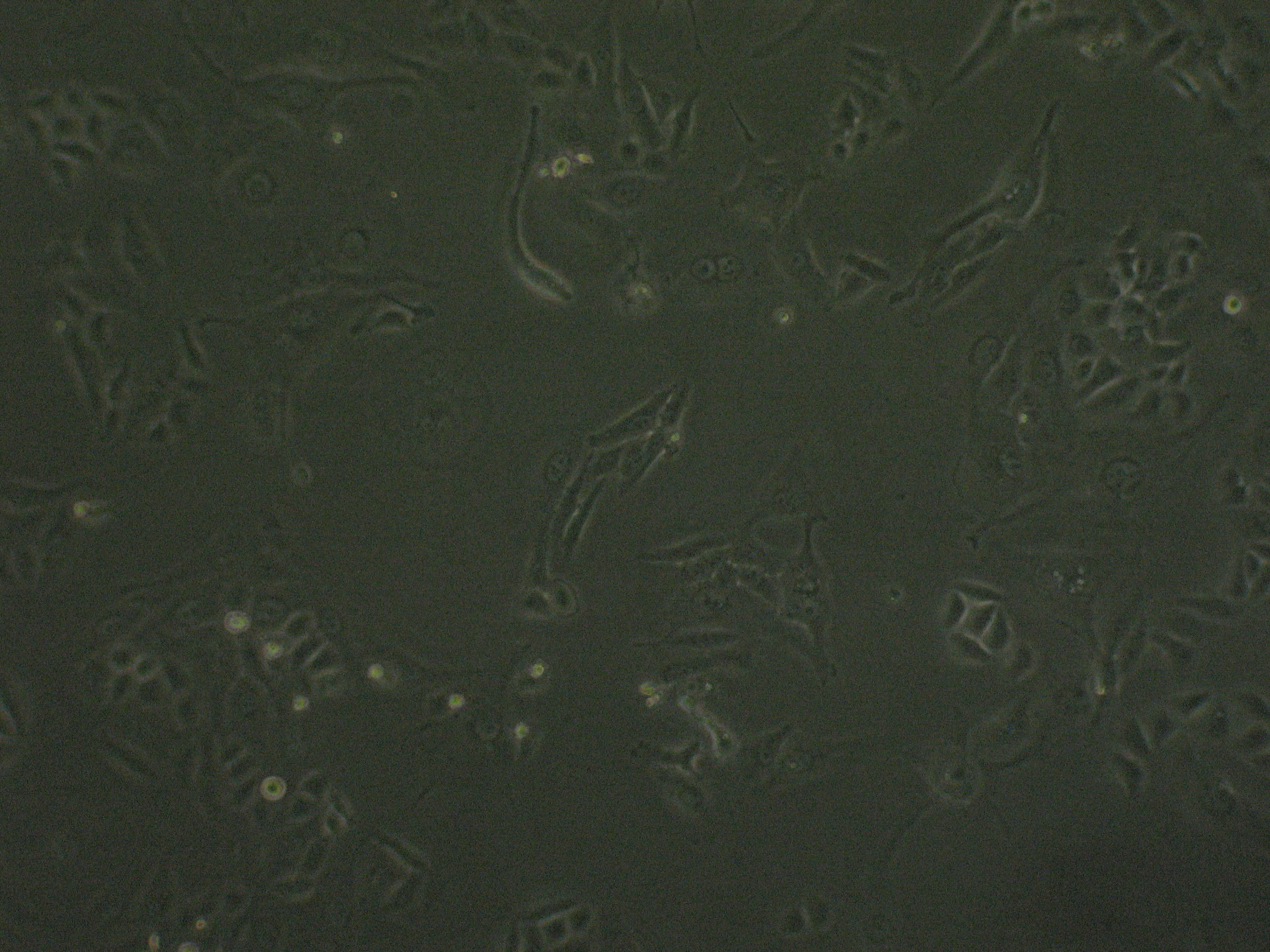

Supplement: S2 File — (ZIP) [file pone.0222896.s002.zip › S2_File/Figure 2A/A549/miR-20a inhibitor.JPG]

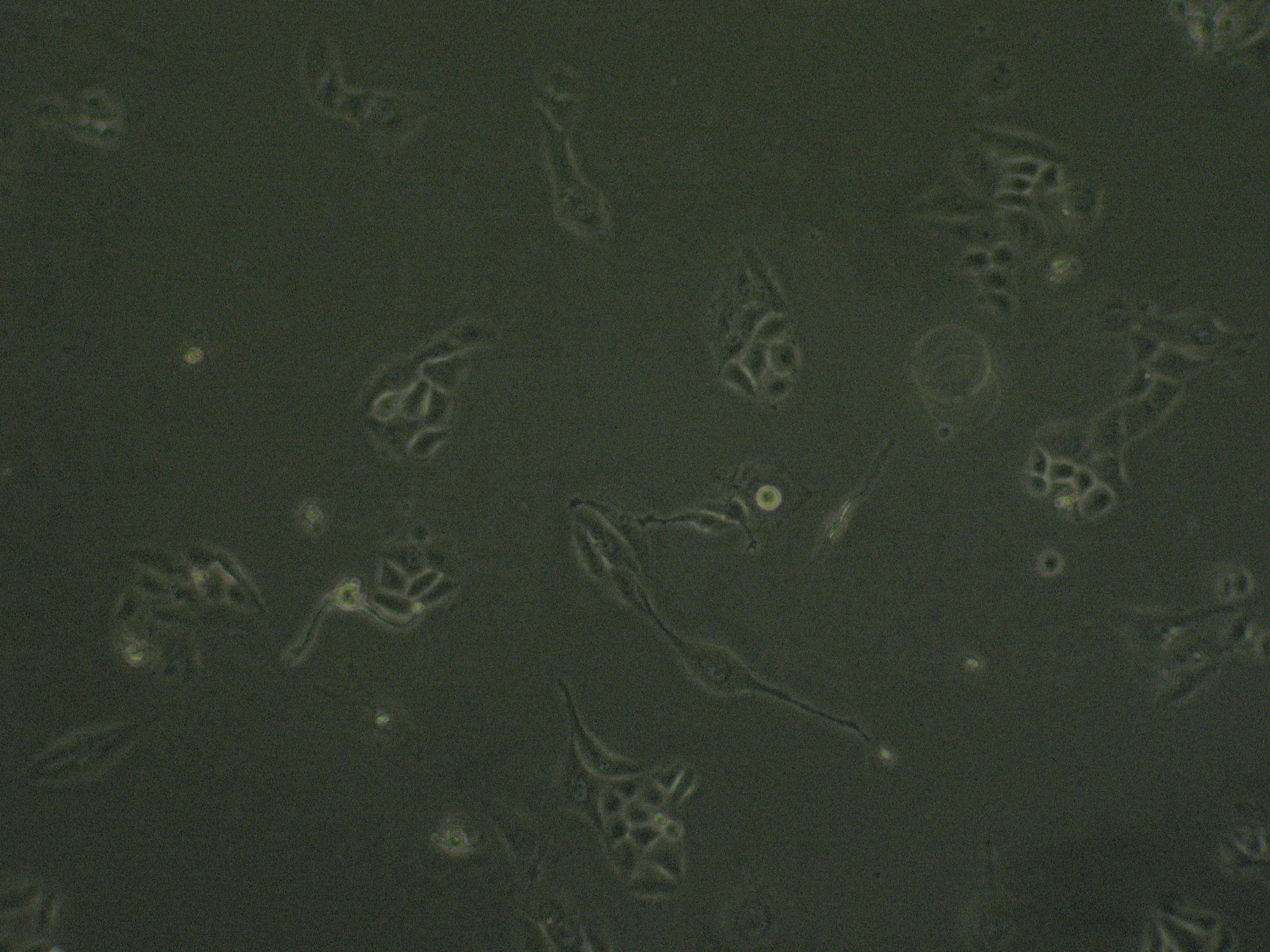

Supplement: S2 File — (ZIP) [file pone.0222896.s002.zip › S2_File/Figure 2A/A549/miR-20b inhibitor.JPG]

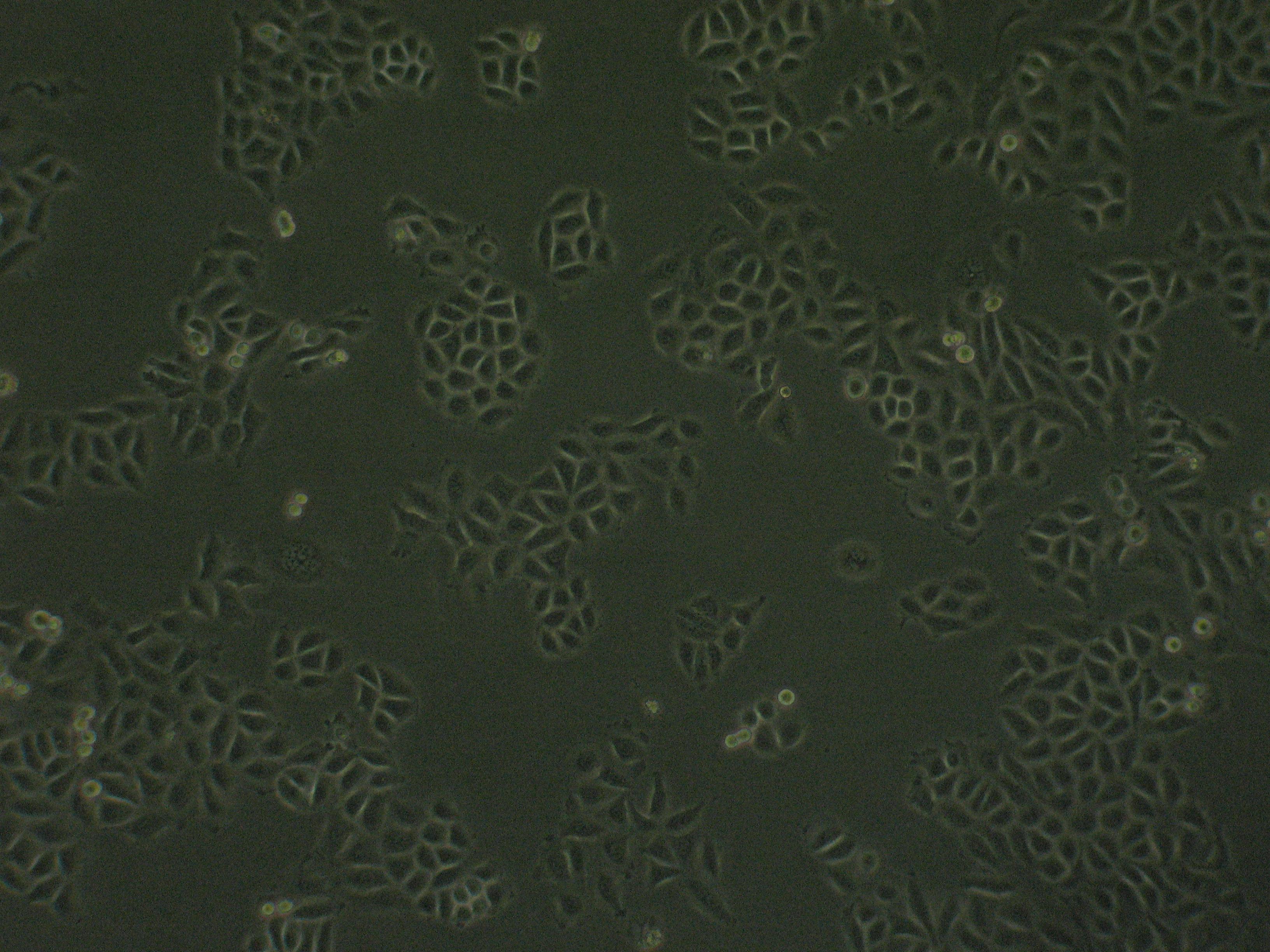

Supplement: S2 File — (ZIP) [file pone.0222896.s002.zip › S2_File/Figure 2A/A549/untreated.JPG]

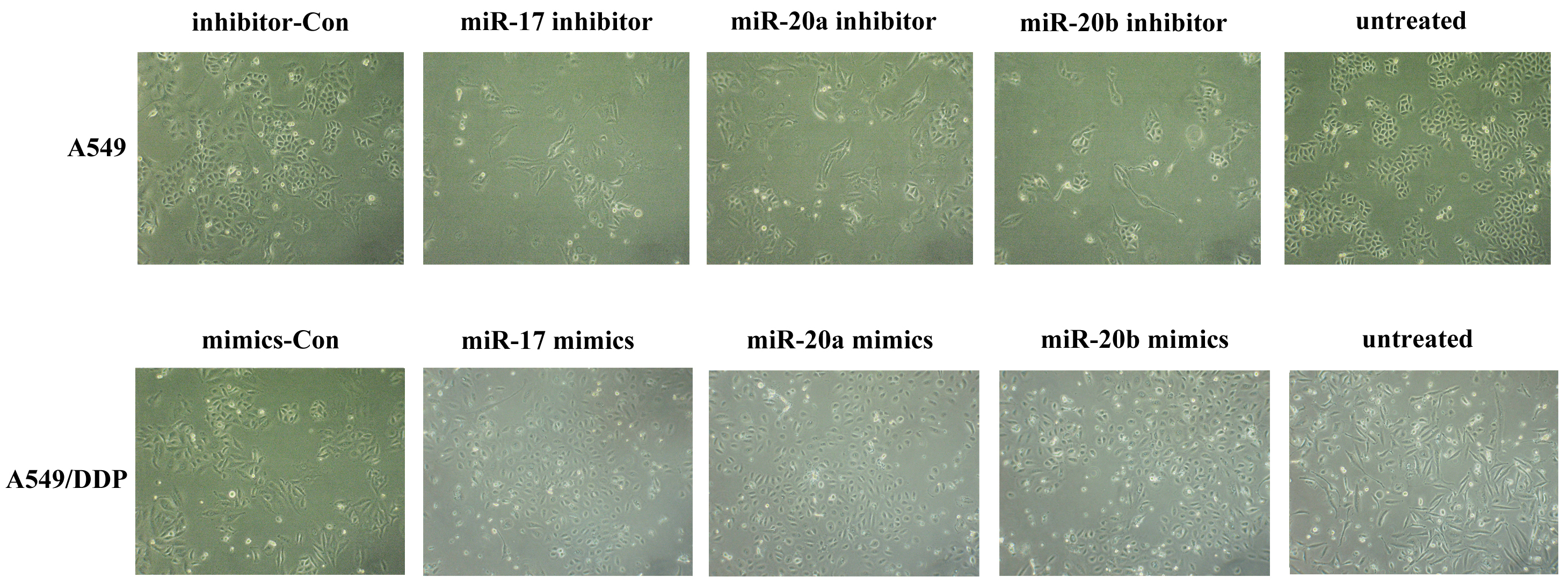

Supplement: S2 File — (ZIP) [file pone.0222896.s002.zip › S2_File/Figure 2A/Figure 2A.jpg]

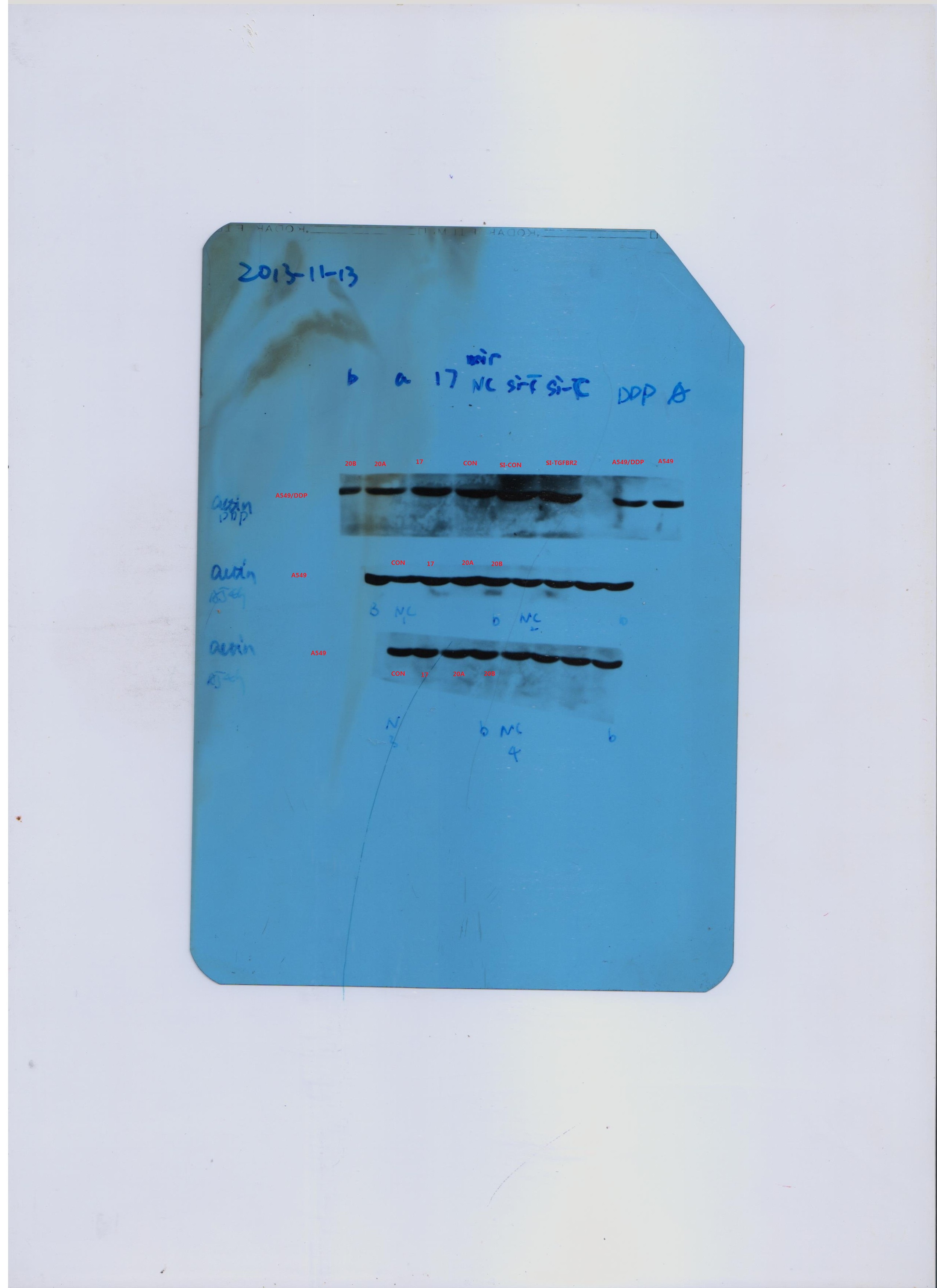

Supplement: S2 File — (ZIP) [file pone.0222896.s002.zip › S2_File/Figure 3C and 4A/3C and 4A Control.jpeg]

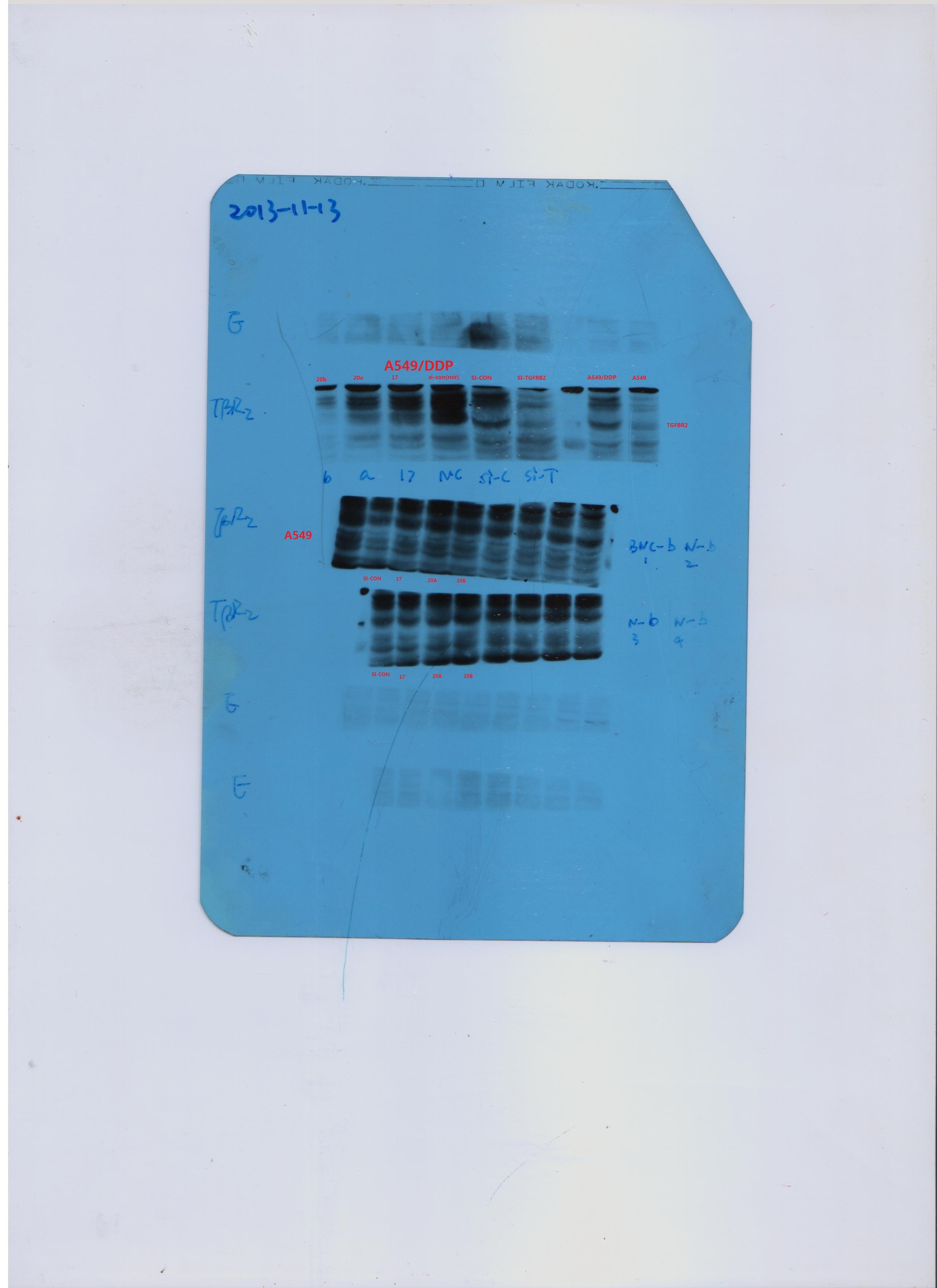

Supplement: S2 File — (ZIP) [file pone.0222896.s002.zip › S2_File/Figure 3C and 4A/3C.jpg]

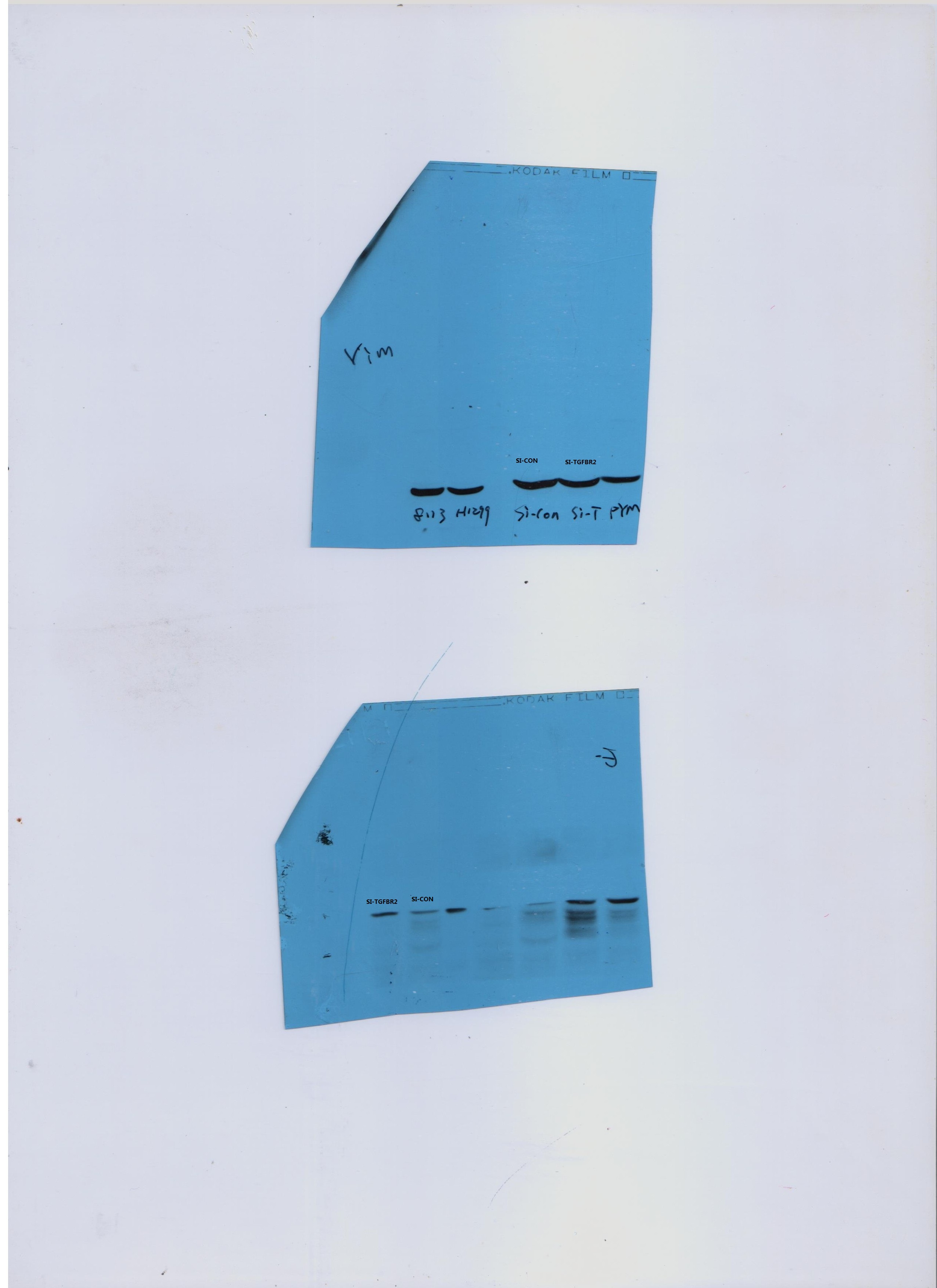

Supplement: S2 File — (ZIP) [file pone.0222896.s002.zip › S2_File/Figure 3C and 4A/4A.jpeg]
